# Clinicopathological analysis of 13 patients with embryonal rhabdomyosarcoma of the female reproductive system in the Chinese population

**DOI:** 10.3389/fonc.2025.1546607

**Published:** 2025-03-14

**Authors:** Liping Bai, Ling Han, Liang Sun, Juan Zou, Yali Chen

**Affiliations:** ^1^ Department of Obstetrics and Gynecology, West China Second University Hospital, Sichuan University, Chengdu, China; ^2^ Key Laboratory of Birth defects and Related Diseases of Women and Children (Sichuan University), Ministry of Education, Chengdu, China; ^3^ Department of Pathology West China Second University Hospital, Sichuan University, Chengdu, China

**Keywords:** embryonal rhabdomyosarcoma, *DICER1*, female reproductive system, clinicopathology, immunohistochemistry

## Abstract

**Objective:**

Examine clinicopathological traits and differential diagnosis of ERMS in female reproductive system.

**Methods:**

Retrospectively assess 13 patients’ data (Jan 2018 - Jun 2024, West China Second Univsity Hospital), covering clinical, histological, immunohistochemical aspects and literature review.

**Results:**

Age 2 months - 67 years (median 21), sites in cervix (5), ovaries (3), uterus (2). Non-specific symptoms. Lesions with grape-like etc. morphologies. Immunohistochemistry: the tumor cells expressed Myogenin (11/13), Desmin (13/13), MyoD1 (12/13) and Myoglobin (5/9). 4/5 had *DICER1* mutations. According to the Children’s Oncology Group Soft Tissue Sarcoma (COG-STS) risk classification, 11 low risk, 2 high risk. Treatments: 8 surgery + chemotherapy, 2 surgery + chemotherapy + radiotherapy, 2 surgery only. 4 died, 8 survived, 1 lost follow up. Follow-up 2 - 41 months (median 20).

**Discussion:**

ERMS is rare, diagnosed by histology and immunohistochemistry, *DICER1* mutation may assist. Treatment is surgery + chemo ± radiotherapy, efficacy related to multiple factors. When ERMS is diagnosed, it is mostly in the early stage, and the treatment method is mostly surgery plus chemotherapy with or without radiotherapy. However, the treatment effect is related to factors such as staging, Intergroup Rhabdomyosarcoma Study (IRS) clinical grouping, COG-STS risk, patient age, and TP53 mutation. There is no clear guideline for the treatment of adult patients.

## Introduction

Rhabdomyosarcoma (RMS) is a rare type of tumor that mostly occurs in children and adolescents and is less common in older adults (1). In the population under 20 years old, the overall incidence rate of RMS is approximately 4.5 cases per million patients (2). Soft tissue sarcomas account for about 3 - 7% of childhood cancers and 1% of adult cancers (1, 3, 4). Approximately half of all childhood soft tissue sarcomas are RMS, which are highly malignant tumors seen as rhabdomyoblasts with varying degrees of differentiation (3, 4). The WHO classification scheme divides rhabdomyosarcoma RMS into four different subtypes: embryonal, alveolar, spindle cell/sclerotic, and pleomorphic subtypes (5) and mentions new subtypes to be studied more, such as rhabdomyosarcoma associated with the EWSR1/FUS::TFCP2 gene fusion and the MEIS1::NCOA2 gene fusion (5, 6). Embryonal rhabdomyosarcoma (ERMS) accounts for 70 - 80% of all RMS diagnoses (5, 7). Although one-third of ERMS cases are diagnosed within 5 years after birth, they can occur at any age, including adulthood. It is worth noting that approximately half of ERMS cases originate from the head and neck region, including the orbit, while the other half occur in the genitourinary system (5, 7). Almost all cervical ERMS and nearly 67% of uterine corpus ERMS carry *DICER1* mutations, while vaginal RMS are mostly *DICER1* wild-type (8). This study retrospectively analyzed the clinical manifestations, treatment, and prognosis of female patients with ERMS in the female reproductive system admitted to West China Second University Hospital, Sichuan University from January 2018 to June 2024, in order to provide clinical evidence for the treatment and prognosis of this type of disease.

## Materials and methods

This retrospective, observational, single-center study was conducted at West China Second University Hospital, Sichuan University, Chengdu, China after obtaining ethical approval from the hospital’s ethics committee. The clinicopathological data of 13 female patients with ERMS in the female reproductive system admitted to West China Second University Hospital, Sichuan University from January 2018 to June 2024 were collected. The study retrospectively examined the patients’ clinical and pathological information. The pathological specimens were independently reviewed by two pathologists from West China Second University Hospital. The clinicopathological data of all 13 patients with ERMS in the reproductive system were reviewed, and a retrospective analysis was performed on their clinical and pathological characteristics, treatment methods, recurrence, post-recurrence treatment, and prognosis. The study collected basic patient information, including age, symptoms, tumor characteristics (such as location and size), surgical methods, comorbidities, adjuvant treatment, recurrence and metastasis rates, follow-up time, and prognosis. In addition, pathological characteristics and *DICER1* mutation test results were also collected. The effectiveness of tumor treatment in patients was evaluated through outpatient follow-up and telephone follow-up.

### Statistical analysis

Statistical analysis was performed using SPSS 27.0 software. Categorical data were presented as the number of cases and percentage (%). Normally distributed continuous data were expressed as mean ± standard deviation (xˉ ± s), while non-normally distributed continuous data were presented as median (range). Survival analysis was carried out using the Kaplan-Meier method, and the log-rank test was used for comparing survival rates. A P-value less than 0.05 was considered statistically significant.

## Results

### Clinical features

Thirteen patients diagnosed pathologically with ERMS in the female reproductive system were included in this study. The basic characteristics of the patients are shown in **Tables 1**, **2**. The ages of the 13 ERMS patients ranged from 2 months to 67 years, with a median age of 21 years, among which 7 patients were adults over 18 years old. The tumors originated from the cervix in 5 cases, from the ovaries in 3 cases, from the uterine body in 2 cases, from the vagina in 1 case, from the vulva in 1 case, and from the pelvic cavity in 1 case. The tumor sizes varied from 3 cm to 21 cm, with a median diameter of 8 cm. Four patients presented with vaginal bleeding (irregular vaginal bleeding and postmenopausal vaginal bleeding), accounting for 30.77%. Four patients presented with masses in the corresponding sites, accounting for 30.77%. Three patients presented with lower abdominal pain, accounting for 23.08%. One patient presented with abdominal distension, accounting for 7.70%. One patient presented with a palpable pelvic mass, accounting for 7.70%. According to the latest International Federation of Gynecology and Obstetrics (FIGO) staging: 6 cases were in stage I, 4 cases were in stage II, 2 cases were in stage III, and 1 case was in stage IV. According to the Intergroup Rhabdomyosarcoma Study Group (IRSG) clinical staging: 11 cases were in stage 1 and 2 cases were in stage 4. In terms of the IRSG clinical grouping, 7 cases were in group I, 1 case was in group II, 3 cases were in group III, and 2 cases were in group IV. According to the Children’s Oncology Group Soft Tissue Sarcoma (COG-STS) risk classification, 9 cases were at low risk, 2 cases were at medium risk, and 2 cases were at high risk. Notably, case 1 of them had a cerebellar medulloblastoma (WHO grade 4) at the age of 4 years and was treated with radiotherapy 31 times after surgical resection of the lesion, with no significant abnormality on regular review; the father of case 7 had a history of gastric cancer. The remaining patients had no family history of tumor at the cutoff of follow-up.

**Table 1:** Clinical Characteristics of ERMS.

Case	Age	Location	Symptoms	Tumor size (cm)	Pathological pattern	FIGO stage	IRSG stage	IRSG clinical groups	COG-STS risk	Surgical procedure	Adjuvant therapy	Recurrence/metastasis/progressive disease	status	Follow up time(months)
1*	11y6m	cervix	Neoplasm	9.4	ERMS	Ib3	1	IIa	low	Lx	CT	No	Disease-free	9
2	16y0m	cervix	Neoplasm	3	ERMS	Ib2	1	Ia	low	Lx+CKC	CT	No	Disease-free	20
3	16y6m	cervix	IVB	10	ERMS	Ib3	1	Ia	low	RH+PLA	CT	No	Disease-free	25
4	46y0m	cervix	IVB	5	ERMS	Ib3	1	Ia	low	RH+PLA	CT	No	Disease-free	30
5	21y8m	Right Ovary	Bloating	21	ERMS	IV	4	IV	high	HYS+BSO+PLA+OMT+AP	CT	No	Disease-free	36
6	54y6m	Uterus	PMVB	4	ERMS	Ib	1	Ia	low	HYS+BSO	ND	No	Disease-free	9
7^#^	48y4m	Cervix	IVB	3	ERMS	Ib2	1	Ia	low	RH+BSO+PLA	CT+RT	No	Disease-free	41
8	2m	Vulva	Neoplasm	5.5	ERMS	II	1	IIIb	low	Lx	CT+RT	Metastasis after 1 month	Died	22
9	42y3m	Uterus	Pelvic Masses	20	ERMS	II	1	Ib	low	Lx	unknown	unknown	Lost to follow up	Lost to follow up
10	1y1m	Vagina	Neoplasm	3	ERMS	IIb	1	IIIb	low	Partial Lx	ND	Progressive Disease	Died	2
11	33y9m	Left Ovary	LAP	10	Embryonal carcinoma+ERMS	III	4	IV	high	HYS+BSO+PLA+OMT+CRS	CT	Metastasis after 5 months(lung and lymph nodes)	Died	17
12	67y5m	Left Ovary	LAP	15	HGSAC+ERMS	IIa	1	Ib	low	HYS+BSO	CT	No	Disease-free	38
13	15y10m	Pelvic	LAP+edeme^&^	8	ERMS	III	1	IIIa	low	Needle biopsy	CT 1 time	Progressive Disease	Died	7

FIGO, The International Federation of Gynecology and Obstetrics; IRSG, Intergroup Rhabdomyosarcoma Study clinical grouping; COG-STS, Children’s Oncology Group -Soft-Tissue Sarcoma; IVB, irregular vaginal bleeding; PMVB, postmenopausal vaginal bleeding; LAP, lower abdominal pain; Lx, Lesionectomy; CKC, cold-knife conization; RH, radical hysterectomy; HYS, hysterectomy; PLA, pelvic lymphadenectomy; BS, bilateral salpingectomy; BSO, Bilateral salpingo-oophorectomy; OMT, omentectomy; AP, appendectomy; CRS, Cytoreductive surgery; HGSAC, High-Grade Serous Adenocarcinoma; PLM, presumed leiomyoma; DYS, dysmenorrhea; CT, Chemotherapy; RT, Radiotherapy; 1*: A medulloblastoma of the cerebellum (WHO Grade 4) was diagnosed at the age of four; 7#: Father has gastric cancer; edeme&:edema of lower extremity.

**Table 2:** Results of immunohistochemistry for the 13 patients with ERMS.

Case	Myogenin	MyoD1	Desmin	Myoglobin	S-100	CK-P	ER	PR	TP53	Ki67(%)	cartilaginous nodules	*DICER1*
1	P+	+	+	F+	-	-	-	-	Wild type	80	-	Mutation of exon 25 c.5437G>A(p.E1813K)
2	P+	+	+	-	-	-	-	-	Wild type	80	-	Mutation(E1813A)
3	F+	+	F+	+	ND	-	ND	ND	ND	65	-	Negative*
4	P+	+	+	F+	-	-	-	-	Wild type	80	-	ND
5	F+	P+	P+	-	ND	ND	ND	ND	ND	60	+	Mutation of codon 1709(D1709N)
6	P+	P+	F+	F+	F+	ND	-	-	Wild type	85	+	Mutation(D1810Y)
7	P+	+	+	F+	ND	ND	ND	ND	Wild type	60	+	ND
8	P+	+	+	ND	ND	-	ND	ND	ND	85	-	ND
9	P+	P+	+	ND	-	ND	-	-	ND	85	-	ND
10	+	+	+	-	ND	ND	-	-	ND	75	-	ND
11	-	?	+	-	-	+	ND	ND	Mutant type	90	-	ND
12	-	+	+	ND	ND	ND	-	-	Mutant type	45	-	ND
13	+	+	+	ND	ND	-	ND	ND	ND	70	-	ND

F+, focal positive; P+, patchy positive; +, positive; -, negative; ND, not done; ?, cannot be clearly defined; Negative*, represents only exon 24 and 25 negatives.

### Pathological features and molecular results

**Figure 1:**
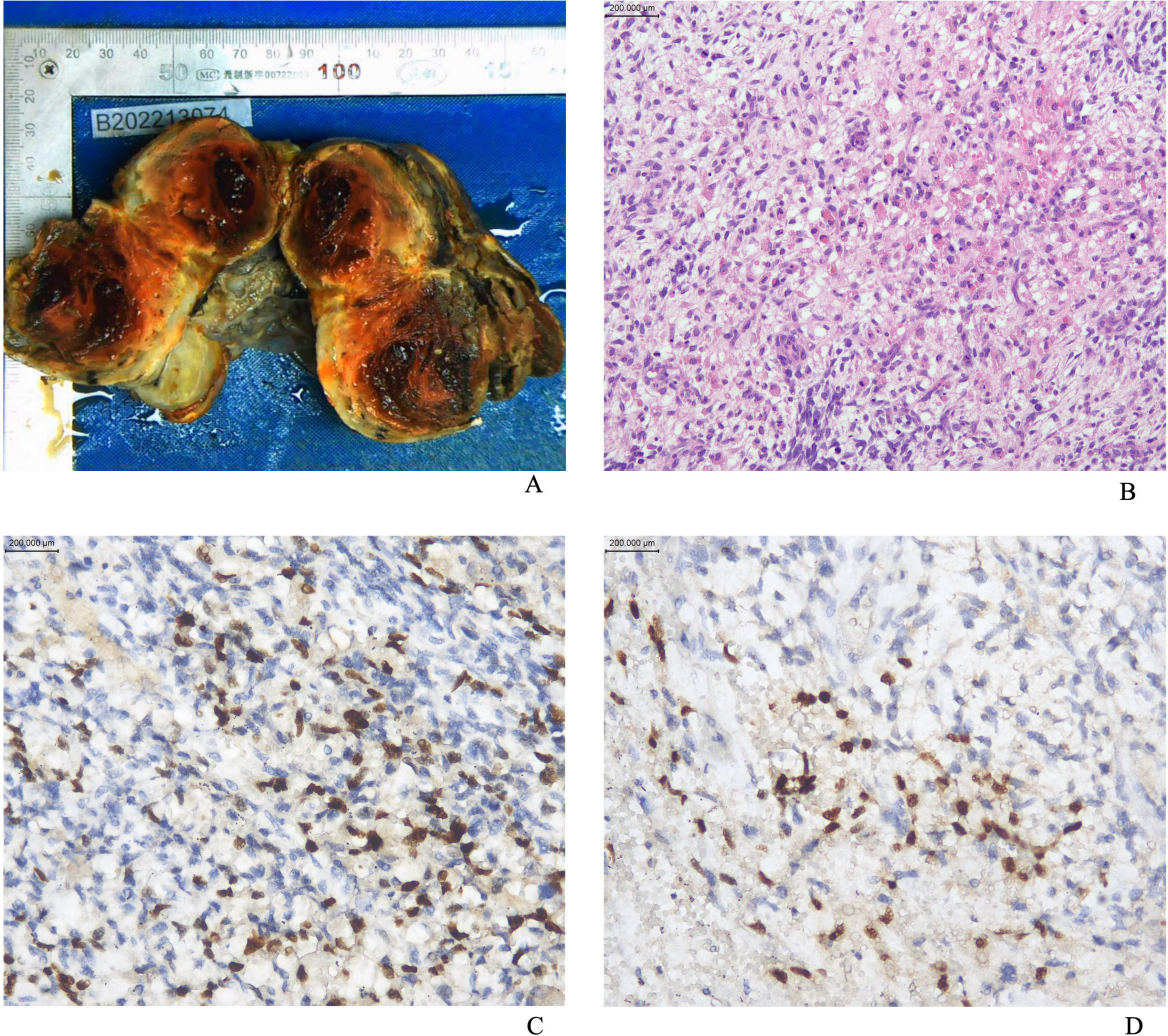
**(A)** The appearance of the gross specimen of ovarian ERMS; **(B)** The image of ERMS stained with hematoxylin and eosin (HE); **(C)** Positive staining for MyoD1; **(D)** Positive staining for Myogenin.

Macroscopically, the lesions were mostly solid or cystic-solid and could be grape-like, polypoid, cauliflower-like or fish-flesh-like. Microscopically, irregular bundles of immature skeletal muscle fibers are seen in a myxoid background. The cells had the characteristics of fetal myotubes, i.e., spindle-shaped outline, central oblong nucleus, and eosinophilic cytoplasm. Cartilaginous nodules were seen in 3 of 13 patients. Immunohistochemical assays, including desmin, myogenin, myoD1 (**Figure 1**), and the cell proliferation marker Ki67, were performed in all 13 patients; the immunohistochemical results are shown in **Table 2**. Among them, 5 patients underwent *DICER1* gene mutation testing, and 4 of them were positive, as shown in **Table 1**. All 13 patients were diagnosed with ERMS, among which Case 11 and Case 12 were mixed tumors. Case 11 was combined with embryonal carcinoma; Case 12 was combined with high-grade serous adenocarcinoma of the ovary.

### Treatment outcomes

Among the 13 patients, 1 patient underwent needle biopsy plus chemotherapy. However, due to intolerance to chemotherapy, the patient did not complete the chemotherapy treatment. As the disease progressed, the survival period was only 7 months. Twelve patients underwent surgical treatment. Among them, 5 patients had lesion resection, and 7 patients had the organ where the tumor was located removed. Among these 7 patients, 5 patients underwent radical surgeries including pelvic lymph node dissection, and 2 patients underwent hysterectomy plus bilateral adnexectomy. Among all the patients, 1 patient did not receive adjuvant treatment, 8 patients only received chemotherapy, and 2 patients received chemotherapy plus radiotherapy. Among the 10 patients who received chemotherapy, 7 patients were treated with the chemotherapy regimen of vincristine + dactinomycin + cyclophosphamide/isocyclophosphamide. One of these patients had a total survival time of 22 months due to disease progression, refractory recurrence, change of chemotherapy regimen, adjuvant radiotherapy, and secondary surgery. One patient with stage IV disease was treated with epirubicin + carboplatin chemotherapy and then albumin-bound paclitaxel + nedaplatin chemotherapy. After 36 months of follow-up, the patient is currently disease-free. One patient with combined embryonal carcinoma was treated with bleomycin + etoposide + cisplatin chemotherapy. After 17 months of follow-up, the patient has passed away. One patient with combined high-grade serous ovarian cancer was treated with paclitaxel + carboplatin chemotherapy plus bevacizumab. After 38 months of follow-up, the patient is currently disease-free. The clinical and pathological characteristics of the patients are shown in **Table 3**.

**Table 3:** Clinical and pathological characteristics of the patients.

Parameter	
Age (years and months )	
Range	2m-67y5m
Median	21y8m
Mean	28y10m
Location	
Cervix	5 (38.46%)
Ovary	3 (23.08%)
Uterus	2 (15.38%)
Symptom	
Vaginal bleeding	4 (30.77%)
Neoplasm	4 (30.77%)
LAP	3 (23.08%)
Size (cm)	
Range	3-21
Median	8
Mean ± SD	8.99 ± 6.23
Follow up months	
Range	2-41
Median	20
Mean ± SD	19.78 ± 13.70
Adjuvant therapy	
CT	8 (61.54%)
CT+RT	2 (15.38%)
IRSG clinical groups	
I、II	8 (61.54%)
III	3 (23.08%)
IV	2 (15.38%)
COG-STS risk	
Low	11 (84.62%)
high	2 (23.08%)
Incidence	
Tumor Progression	2 (15.38%)
Metastasis	2 (15.38%)
Died	4 (30.77%)

CT, Chemotherapy; RT, Radiotherapy; IRSG, Intergroup Rhabdomyosarcoma Study Group; COG-STS, Children’s Oncology Group Soft-Tissue Sarcoma.

**Figure 2:**
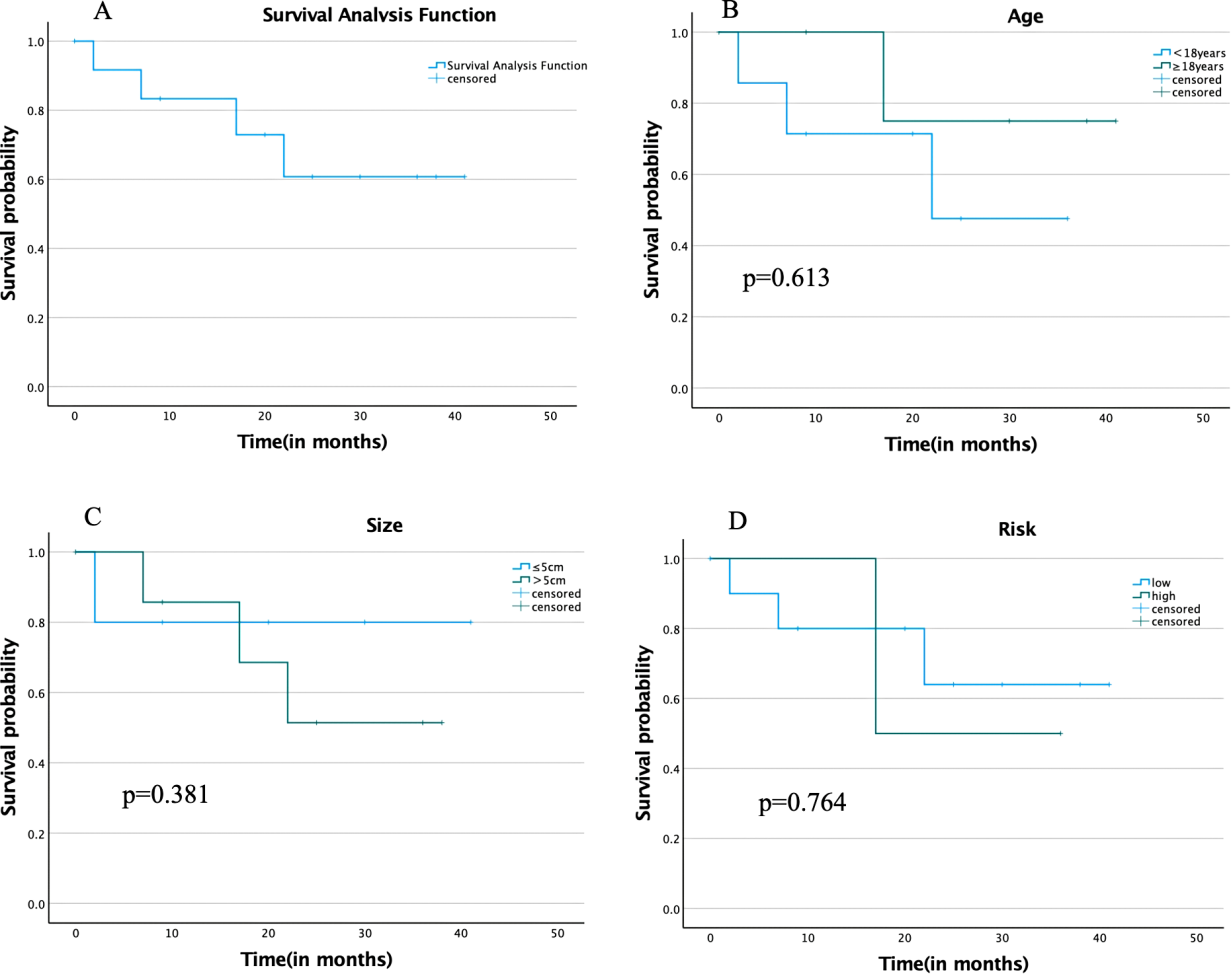
**(A)** Overall survival analysis; **(B)** Survival analysis by different age groups; **(C)** Survival analysis by different tumor size groups; **(D)** Survival analysis by different degrees of risk in COG-STS.

During the follow-up period, of the 13 patients, 8 survived tumor-free, 4 died, and 1 was lost to follow-up. Disease progression was observed in 2 cases during the follow-up period, recurrence of metastasis in 2 cases, and death in all 4 patients. The follow-up period ranged from 2 months to 41 months, with a median follow-up period of 20 months and a 75th percentile survival time of 17 months, and the survival analysis is shown in **Figure 2**.

## Discussion

ERMS is named because it resembles skeletal muscle in embryonic development (9). ERMS is rare and difficult to diagnose. In the female reproductive system, ERMS is commonly found in the cervix and vagina. It usually presents as a polypoid mass or multiple polyps (botryoid). Histopathologically, embryonal rhabdomyosarcomas contain primitive mesenchymal cells at different stages of myogenesis. Typical embryonal rhabdomyosarcoma consists of rhabdomyoblasts with different degrees of differentiation in sparse mucus-like mesenchyme with alternating areas of dense and sparse cell density (5). The cells have small ovoid hyperchromatic nuclei and a small amount of cytoplasm, and there are often cartilaginous nodules. The diagnostic criteria for ERMS is the presence of cambium layer. Immunohistochemical markers include myogenic markers (diffuse desmin positivity, and MyoD1 and myogenin may be focally positive), and hormone receptors are often negative (8). The molecular biological characteristics are that cervical embryonal rhabdomyosarcoma and uterine corpus embryonal rhabdomyosarcoma often carry *DICER1* mutations (8, 10, 11).

ERMS of the uterus usually need to be differentiated from adenosarcoma, and the morphological overlap between ERMS and adenosarcoma makes it difficult to distinguish between the two tumors. Pathological features of adenosarcomas: biphasic tumors, composed of benign epithelial and malignant mesenchymal components; usually polypoid lesions, histologically with a low-grade lobular structure, similar to lobular tumors of the breast, with glands lined by benign Müllerian epithelium, and mesenchymal stroma usually of low-grade spindle cells, which can present with differentiation of high-grade heterogeneous components (most commonly skeletal muscle) and sarcomatous overgrowth (8).On immunohistochemical testing, low-grade adenosarcomas are usually positive for CD10 and hormone receptors (8).

Heterozygous germline mutations of *DICER1* were first discovered in 2009 in a series of children with pleuropulmonary blastoma (PPB) (12). The Dicer protein encoded by the *DICER1* gene is an endoribonuclease that participates in the production of small RNAs such as microRNA and small interfering RNA and is crucial for the regulation of gene expression during development (10, 11, 13). The germline pathogenic variants that lead to *DICER1* syndrome are usually truncating alterations (such as nonsense, frameshift, or splice - site mutations), which result in a loss of function; while the pathogenic somatic mutations are almost entirely missense substitutions that affect the five “hot spots” in the RNAse IIIb domain. These mutations change the enzyme’s ability to process microRNA, leading to an abnormal mix of microRNAs (10, 11, 13).

Almost all cervical ERMS and nearly 67% of uterine corpus ERMS carry *DICER1* mutations, whereas vaginal ERMS are *DICER1* wild-type (10, 14–16). Therefore, when cervical ERMS is suspected, deletion of *DICER1* mutations often suggests incompatibility with this diagnosis. On the other hand, since *DICER1* mutations are present in 26% to 42% of adenosarcomas (14, 17), the presence of *DICER1* mutations does not differentiate ERMS from adenosarcoma. In ERMS with *DICER1* mutations, genetic counseling is necessary to investigate the possibility of *DICER1* syndrome. One of the 13 patients we analyzed suffered from cerebellar medulloblastoma (WHO grade 4) at the age of 4 years; in another case, the father had a history of gastric cancer. The remaining patients had no significant family history of the tumor, and none of the patients underwent genetic counseling. Five of these 13 patients underwent *DICER1* genetic testing, and mutations were detected in four of them (two cervical, one ovarian, and one uterine). Another patient with cervical ERMS did not detect mutations in the *DICER1* gene in exons 24 and 25, which may be related to an insufficient number of tested loci.

Currently, surgery + chemotherapy ± radiotherapy is the recommended treatment modality for ERMS (18). For children and young women, initial treatment is preferred to surgery that can completely remove the tumor (resection should be 0.5 cm beyond the tumor margins) while preserving as much organ function as possible, while reproductive adults tend to undergo surgical procedures with more complete organ removal, such as hysterectomy (19–21). Surgery that simply reduces the size of the tumor and does not completely resect the tumor is not superior to biopsy in terms of improving prognosis (19).The Children’s Oncology Group (COG) and the European Pediatric Soft Tissue Sarcoma Study Group (EpSSG) use different chemotherapy-based regimens, the main difference being the choice of alkylating agent, with the COG using cyclophosphamide and the EpSSG using isocyclophosphamide. Comparison of these two alkylating agents shows that they do not differ significantly in therapeutic efficacy but produce different long-term side effects: isocyclophosphamide is more nephrotoxic, whereas cyclophosphamide is more gonadotoxic (22, 23).In adults, because of the rarity of RMS, chemotherapy regimens are mostly based on the selection of drugs based on experience with pediatric RMS. The response rate to chemotherapy for adult embryonal rhabdomyosarcoma is approximately 85% (1). Radiotherapy is an important part of the comprehensive treatment of RMS in children, especially for those patients with inoperable resection, microscopic residual tumor, naked eye residual tumor, or lymph node involvement, induction chemotherapy followed by simultaneous radiotherapy is the currently recommended treatment modality (19).

IRSG combines staging, clinical grouping, and pathology type to categorize RMS into low-risk, intermediate-risk, and high-risk groups. The 5-year EFS of RMS in patients in the low-risk, intermediate, and high-risk groups were 87%-90%, 65%-73%, and <30%, respectively (24), and this system was able to better predict the prognosis of patients, which is instructive for the selection of treatment options. In addition to influencing factors such as tumor stage, subgroups, and risk level grading, *FOXO1* fusion positivity, age less than 1 year, age greater than 10 years, and TP53 mutations are poor prognostic factors (25–29). Higher levels of TP53 protein have been found in metastatic ERMS relative to limited ERMS (30). 95% of embryonic rhabdomyosarcomas are *FOXO1* fusion-negative, which means that almost all embryonic rhabdomyosarcomas are *FOXO1* fusion-negative rhabdomyosarcomas (31). ERMS is genetically free of gene fusions, but aneuploidy and multiple genetic alterations are present; ERMS has an overall favorable prognosis, but cases with diffuse interstitial changes have a poorer prognosis (6, 31, 32).

Adult patients with RMS have a lower overall survival rate than pediatric patients, and the few published series on adult patients describe a poorer prognosis, with 5-year survival rates ranging from 20-51.8% (33–36). Even with the same tissue type, site, and stage, adults still have a worse prognosis than children (35). There is an increasing number of studies related to RMS in adults (18, 26, 36–39), and the reasons for the currently reported poorer survival in adult patients may include delayed diagnosis, health system disparities, increased expression of multidrug-resistant proteins in tumors, and low tolerance to intensive therapy (18, 26, 36, 37), but also because of variations in the distribution of sarcoma subtypes and clinical behaviors in different age groups, racial differences, or biological differences (37–39). One study found that the survival of patients with embryonic and alveolar RMS in an Asian population was inferior to that previously reported in other races (39), suggesting that tumor heterogeneity may exist between different races. In another study, multiple chemoresistance genes were found to be upregulated in adult RMS patients, and pharmacological analyses showed that anthracycline-based regimens had the highest sensitivity to tumor cells in both 2D and 3D culture systems, suggesting that anthracyclines may be promising agents (37).

Four of the 13 patients reported in this article died, three of whom were minors and one an adult. Of the three minors who died, two died because they did not complete treatment and their disease progressed; one patient with vulvar ERMS who was less than 1 year old, who had a recurrence in the first month postoperatively, changed chemotherapy regimens, supplemental radiotherapy, and had a second surgery, still died at 22 months postoperatively. The other four minor patients who completed treatment survived tumor-free. The prognosis of the patients who completed treatment was consistent with the literature. Among the adult patients, one adult patient with combined embryonal carcinoma developed lung metastasis and lymph node metastasis 5 months after surgery and died 17 months after surgery. Of the remaining adult patients, 5 survived tumor-free and 1 was lost to follow-up. Of note is the case of a 21-year-old patient with ovarian ERMS, stage IV high-risk, who remained tumor-free and survived 36 months postoperatively, probably thanks to the patient’s residual-free surgical treatment and removal of metastatic lesions in the abdominopelvic cavity.

In this article, 7 of our 13 cases were adult ERMS patients, which can provide data for the study of adult rhabdomyosarcoma. However, our study has some limitations, such as a small number of cases, a low percentage of patients with genetic testing, a short follow-up period, and a lack of data on genetic counseling. For diagnostic treatment and stratified management of adults, more clinical data need to be collected and studies need to be analyzed.

## Conclusion

ERMS of the female reproductive system is a rare malignant tumor, especially rare in adults, and is challenging to diagnose and treat. Surgery supplemented with chemotherapy, with or without radiation therapy, is the mainstay of treatment. Tumor size, tumor site, presence of metastases, and the completeness of surgery affect the patient’s prognosis. Of all rhabdomyosarcomas, embryonic rhabdomyosarcomas have a relatively good prognosis, but adult patients have a poorer prognosis. In patients with *DICER1* and other gene mutations, the possibility of a tumor syndrome should be considered and genetic counseling should be done. In RMS, age-specific heterogeneity, race-specific heterogeneity, and new tissue molecular subtypes are still being studied and refined. More research is still needed on treatment strategies for adult rhabdomyosarcoma patients with poor prognosis.

## Data Availability

The original contributions presented in the study are included in the article/supplementary material. Further inquiries can be directed to the corresponding authors.
